# Multi-cohort analysis unveils novel microbial targets for the treatment of hyperuricemia and gout

**DOI:** 10.1128/msystems.01091-25

**Published:** 2025-09-17

**Authors:** Jinlong Qie, Man Cao, Min Xu, Yingjie Zhang, Liangen Luo, Chuqing Sun, Dongxian Ke, Songjian Yuan, Wenting Jia, Tianhua Qiu, Tianhua Li, Xiaoman Du, Chuanxing Xiao, Zhenqiang Hong, Bangzhou Zhang

**Affiliations:** 1 School of Pharmacy, Fujian University of Traditional Chinese Medicine 47858, Fuzhou, China; 2 School of Traditional Chinese Medicine, Fujian University of Traditional Chinese Medicine 47858, Fuzhou, China; 3 Key Laboratory of Orthopedics & Traumatology of Traditional Chinese Medicine and Rehabilitation, Ministry of Education 127394, Fuzhou, China; 4 Xiamen Treatgut Biotechnology, Co., Ltd., Xiamen, China; 5 Key Laboratory of Traditional Chinese Medicine Bone Injury and Sports Rehabilitation, Ministry of Education 127394, Fuzhou, China; 6 School of Medicine, Xiamen University 12466, Xiamen, China; LifeMine Therapeutics, Cambridge, Massachusetts, USA

**Keywords:** hyperuricemia, gout, gut microbiota, uric acid, uric acid metabolic gene cluster

## Abstract

The gut microbiota plays a crucial role in the development of hyperuricemia (HUA) and gout. However, the variability in study designs and analytical methods has led to inconsistent conclusions across different studies. Here, we conducted a comprehensive analysis of the gut microbiota associated with HUA and gout by examining 368 16S rRNA sequencing data from four Chinese cohorts, including 159 healthy controls (HC), 136 HUA patients, and 73 gout patients. Our findings indicate that there were significant differences in the gut microbiota composition between the three groups. Specifically, the HUA and gout groups demonstrated an increased abundance of pro-inflammatory bacteria, such as *Fusobacterium* and *Bilophila*, while beneficial bacteria known for their anti-inflammatory properties and metabolic benefits, including *Christensenellaceae* R-7 group, *Anaerostipes,* and *Collinsella*, are relatively reduced. Additionally, we developed a predictive model using microbial markers that achieved a high accuracy (area under the curve [AUC] > 0.8) in distinguishing between the HC, HUA, and gout groups. Notably, further metagenomic analysis identified a species-level genome bin (SGB), designated as *Phil1 sp00194085*, belonging to the order *Christensenellales*. For the first time, we discovered that this SGB carries a uric acid metabolic gene cluster and possesses enzymes associated with purine metabolism, suggesting its potential role in uric acid metabolism. Overall, our study deepens the understanding of the gut microbiota’s role in HUA and gout and lays a foundation for developing innovative therapeutic strategies to effectively control uric acid levels through gut microbiota modulation.

In this study, we conducted a comprehensive analysis of gut microbiota across multiple cohorts, identifying distinct microbial signatures in healthy controls, hyperuricemia (HUA), and gout patients. We observed an increase in pro-inflammatory bacteria and a decrease in beneficial bacteria for host metabolism in both the HUA and gout groups. Additionally, we developed a predictive model with high accuracy (area under the curve [AUC] > 0.8) based on microbial markers and discovered a novel species with potential for uric acid metabolism, providing new therapeutic targets for HUA and gout.

## INTRODUCTION

Hyperuricemia (HUA) is a prevalent metabolic disorder characterized by persistently elevated uric acid levels in the blood. Uric acid, the end product of purine metabolism, originates from endogenous synthesis and dietary intake (1). Approximately 70% of daily uric acid production is excreted renally, while 30% undergoes intestinal bacterial degradation (2). Elevated uric acid levels, caused by overproduction (e.g., aberrant purine metabolic enzymes) or underexcretion (e.g., impaired renal transport proteins), can lead to HUA, gout, nephrolithiasis, and cardiovascular diseases (3). In China, the incidence of HUA has reached 14%, with a trend toward younger populations (4). Current pharmacotherapies include xanthine oxidase inhibitors (allopurinol, febuxostat) and uricosurics (benzbromarone) (5), but their long-term use faces limitations in safety, efficacy, adherence (6), and potential cardiovascular risks (7). Therefore, there is an urgent need for the exploration and development of safer and more effective therapies to lower uric acid levels for patients with HUA and gout.

Recent evidence positions the gut as an important uric-acid elimination route, handling up to one-third of daily output (8). The stability of the gut microbiota is essential for maintaining blood uric acid levels (9). Compared with healthy controls (HC), both HUA and gout patients display marked dysbiosis: lower gene richness and diversity expansion of opportunistic taxa (e.g., *Ruminococcus gnavus*), and depletion of butyrate producers (*Roseburia hominis*, *Odoribacter splanchnicus*, *Butyricimonas synergistica*) (10, 11). Additionally, the gout group was enriched with xanthine dehydrogenase that degrades purines into uric acid, while the activity of uricase that degrades uric acid into urea was reduced, leading to abnormal accumulation of uric acid (11).

Given the close association between the gut microbiota and HUA, modulating the gut microbiome is emerging as a promising treatment for patients with HUA and gout (12, 13). Interventions such as probiotics, prebiotics, and fecal microbiota transplantation have demonstrated potential efficacy in this field (14, 15). For instance, *Lactobacillus plantarum* FS4722 has been found to reduce xanthine oxidase activity, promoting uric acid catabolism and excretion (16). Similarly, *Alistipes indistinctus* has been shown to enhance uric acid excretion mediated by the urate transporter ABCG2 through the production of hippurate, thereby alleviating HUA (17). A recent study identified a conserved gene cluster prevalent in uric acid-consuming gut bacteria, which encodes uric acid degradation pathways, converting uric acid into xanthine or short-chain fatty acids (18). However, the identity of other gut bacterial species potentially capable of regulating uric acid metabolism and their underlying mechanisms remains unclear. Some studies have attempted to use microbial markers to build predictive models that differentiate between HC and individuals with HUA or gout (11, 19). However, these models often rely on single cohorts, limiting their generalizability. To address this, a more comprehensive analysis across multiple cohorts is needed to minimize study heterogeneity and identify reliable microbial markers. This approach is essential for pinpointing precise microbial targets for the treatment of HUA and gout.

In this study, we integrated 368 16S rRNA sequencing data from four Chinese cohorts (20–22), including HC, HUA patients, and gout patients. Using standardized preprocessing methods and batch effect correction, we performed an in-depth analysis of the compositional and functional differences in the gut microbiota across the three groups. This analysis uncovered dynamic changes in microbial communities and identified gut microbial biomarkers that can effectively differentiate HC from those with HUA and gout. Furthermore, through metagenomic analysis of a gout cohort (19), we precisely identified species-level genome bins (SGBs) carrying uric acid metabolic gene clusters and discovered a SGB belonging to the order *Christensenellales* that carries a uric acid metabolic gene cluster. This SGB encodes enzymes linked to purine metabolism, indicating its potential involvement in uric acid regulation. Together, our findings provide new insights for developing innovative treatment strategies for HUA and gout.

## RESULTS

### Multi-cohort analysis reveals significant changes in the structure and composition of the gut microbiota in patients with HUA and gout

**Fig 1:**
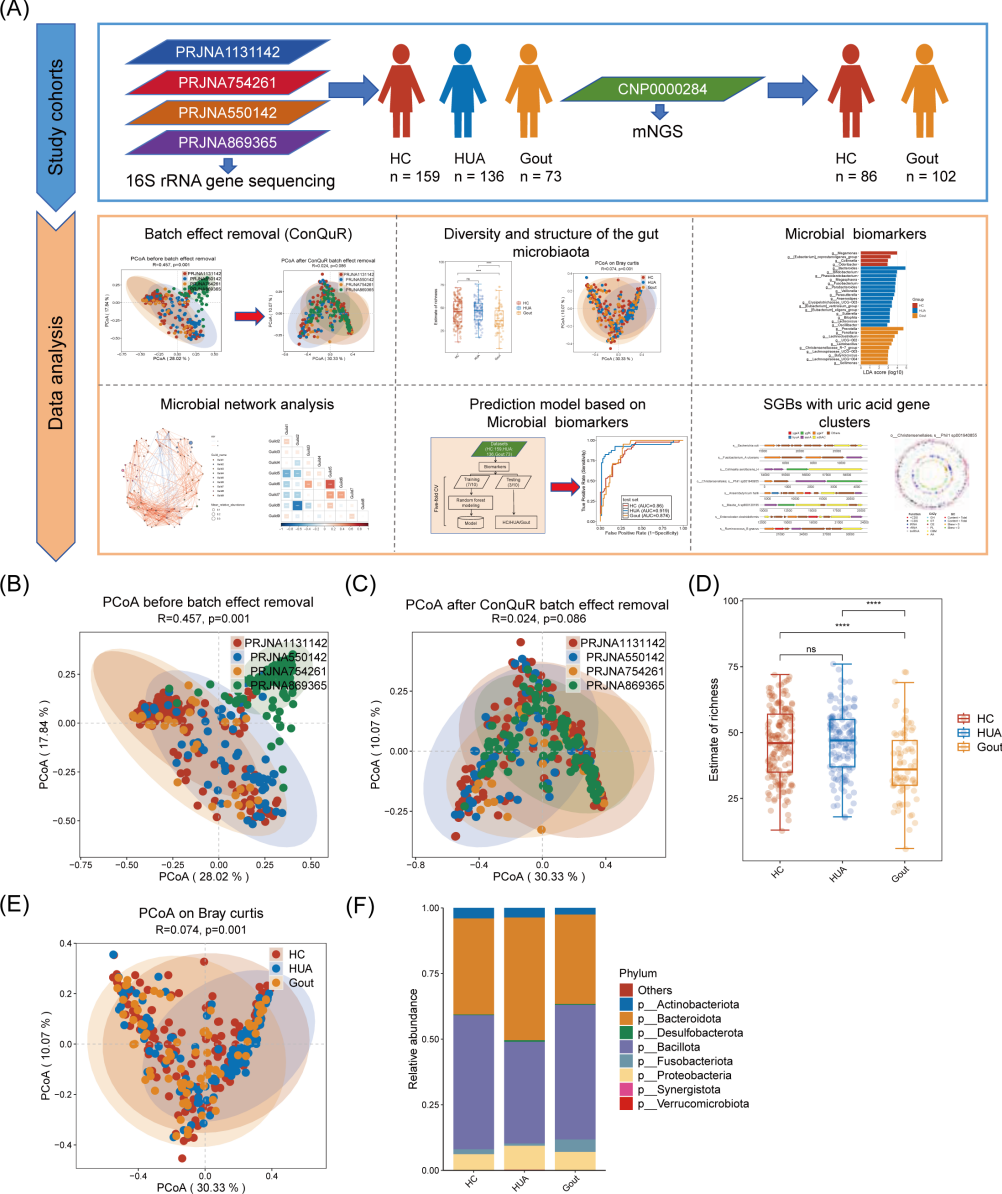
Comparison of gut microbiota diversity and structure among the HC, HUA, and gout groups. (**A**) Overview of the workflow for our data analysis procedure. (**B**) Principal coordinates analysis (PCoA) plot shows all samples using genus-level Bray-Curtis dissimilarity, colored by cohorts before batch-effect removal. The 95% confidence ellipses were shown for samples of different cohorts. Analysis of similarities (ANOSIM), *R* = 0.457, *P* = 0.001. (**C**) PCoA plot shows all samples using genus-level Bray-Curtis dissimilarity colored by cohorts after batch-effect removal by the R package Conditional Quantile Regression (ConQuR). ANOSIM, R = 0.024, *P* = 0.086. (**D**) Alpha diversity indices of genera among the three groups according to the estimate of richness. Significance was determined using the Wilcoxon rank-sum test. ****P < 0.0001; ns for not significant. (**E**) PCoA plot shows all samples using genus-level Bray-Curtis dissimilarity colored by HC, HUA, and gout groups. ANOSIM, *R* = 0.074, *P* = 0.001. (**F**) Pie charts show the proportion of microbiota abundance in the three groups at the phylum level, with different colors corresponding to different phyla. HC, healthy controls; HUA, patients with hyperuricemia.

To delineate the gut microbiota in patients with elevated uric acid levels, we systematically compiled data from four published Chinese 16S rRNA sequencing data sets (Fig. 1A; Table S1). These study cohorts included 159 HC, 136 HUA patients, and 73 gout patients. With the compiled data, our goal is to pinpoint key gut microbes influencing uric acid levels and to develop a predictive model (Fig. 1A; Table S2).

Briefly, we employed standardized bioinformatic preprocessing for raw 16S rRNA sequencing data and detected batch effects across different cohorts' microbiome data (analysis of similarities [ANOSIM], *R* = 0.457, *P* = 0.001; Fig. 1B). To address this, we applied the Conditional Quantile Regression (ConQuR) method to adjust for batch effects. Principal coordinates analysis (PCoA) demonstrated a reduction in heterogeneity among different cohorts (ANOSIM, R = 0.024, *P* = 0.086; Fig. 1C). We further assessed bacterial alpha diversity using richness estimates and the Shannon index. While the Shannon index showed no significant differences among the three groups, the gout group exhibited a lower richness estimate compared to the HC and HUA groups (Fig. 1D; Fig. S1A), consistent with previous results (11). PCoA based on genus-level Bray-Curtis dissimilarity revealed clear distinctions in microbial community structure among the three groups (ANOSIM, *R* = 0.074, *P* = 0.001; Fig. 1E). Additionally, recognizing the Bacillota/Bacteroidetes ratio as a key indicator of gut microbiota structure (23), we compared this ratio across the three groups. Notably, the HUA group exhibited a lower proportion of Bacillota and a higher proportion of Bacteroidetes, resulting in a significantly reduced Bacillota/Bacteroidetes ratio (Fig. 1F; Fig. S1B). Collectively, these results suggest significant alterations in the gut microbial community structure and composition in HUA and gout groups.

### Differential analysis identified key microbial and functional biomarkers in patients with HUA and gout

**Fig 2:**
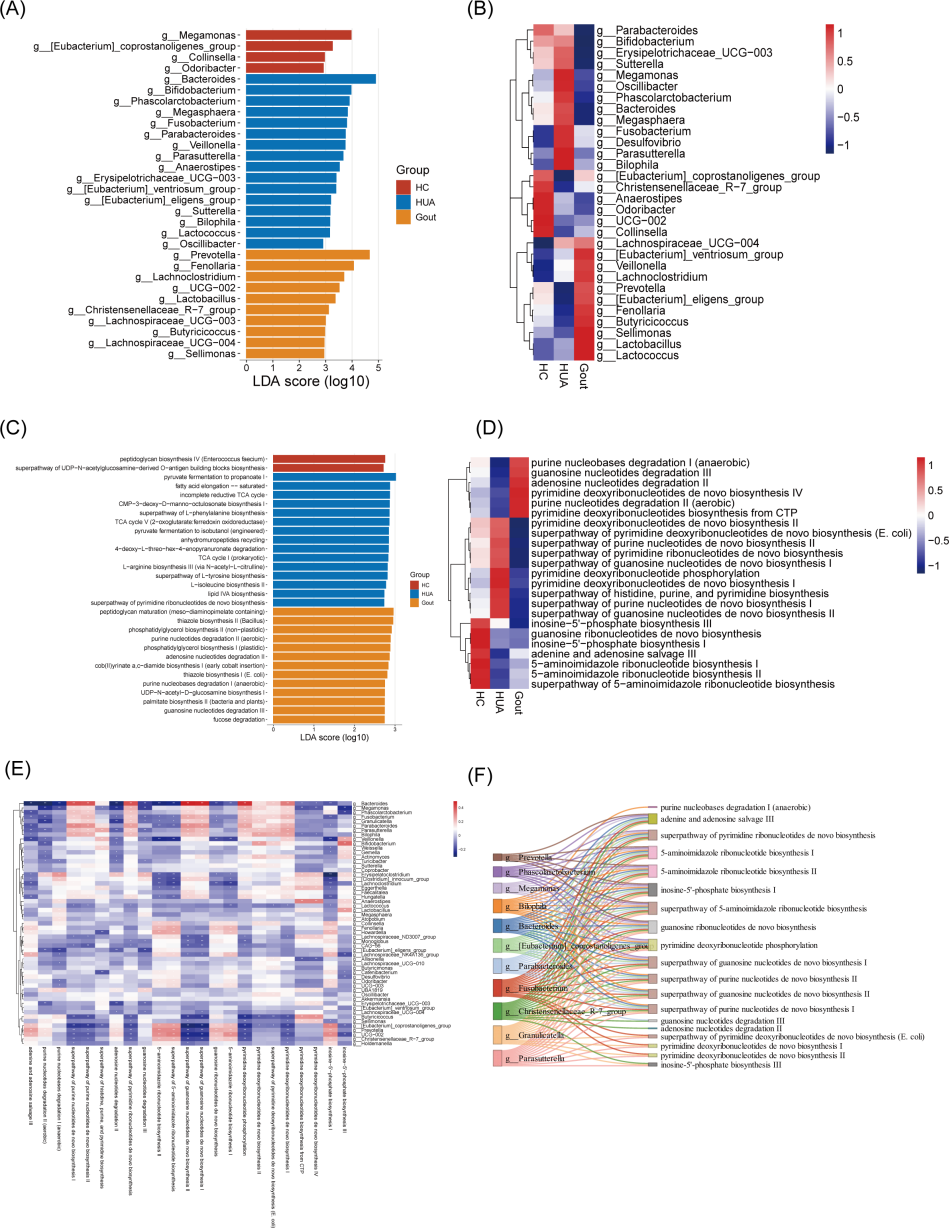
Comparison of gut microbiota composition and function among the HC, HUA, and gout groups. (**A**) The histogram represents linear discriminant analysis (LDA) scores of genera among the three groups identified by LEfSe analysis. Only the top 30 significantly different genera (LDA > 2; Kruskal-Wallis test, *P* < 0.05) were shown. (**B**) Heatmap shows the mean bacterial relative abundance of three groups at the genus level. Only the genera presented in panel A were displayed. (**C**) The histogram represents LDA scores of MetaCyc pathways among the three groups identified by LEfSe analysis. Only the top 30 significantly different MetaCyc pathways (LDA > 2; Kruskal-Wallis test, *P* < 0.05) were shown. (**D**) Heatmap shows the mean relative abundance of three groups at the MetaCyc pathway level. Only the MetaCyc pathways associated with uric acid metabolism were shown with significant differences among the three groups. (**E**) The Spearman correlations between the genera (presented in Fig. 2B) and MetaCyc pathways (presented in panel D). Red: positive correlation (*r* > 0); purple: negative correlation (*r* < 0). *P < 0.05, **P < 0.01, and ***P < 0.001. (**F**) Sankey diagram shows the associations between the genera (presented in panel B) and MetaCyc pathways (presented in panel D) using PICRUSt2.

Given the differences in gut microbiome structure and composition observed in HUA and gout patients, we next conducted a linear discriminant analysis effect size (LEfSe) to identify gut microbial taxa specifically associated with these groups. In total, we identified 58 taxonomic biomarkers with significant differences in abundance across the three groups (Table S3). The top 30 most significantly differing genera are illustrated in Fig. 2A and B. Notably, the HC group showed an enrichment of *Christensenellaceae* R-7 group, *Anaerostipes,* and *Collinsella*, which are recognized for their anti-inflammatory effects and their potential to enhance host metabolism (24–26). In contrast, pro-inflammatory bacteria such as *Fusobacterium* and *Bilophila* were more prevalent in the HUA and gout groups. In addition, *Lactobacillus* and *Lactococcus*, enriched in the gout group, may produce lactic acid that could affect renal excretion of uric acid (27). These results were confirmed by the ANCOM-BC2 method (28), with particular emphasis on the reduction of the *Christensenellaceae* R-7 group in the HUA and gout groups, indicating its key role in uric acid regulation (Fig. S2; Table S4).

Considering the impact of the gut microbiota metabolism on uric acid levels, we applied PICRUSt2 software to predict the functional profiles of the gut microbiota in the HUA and gout groups at the MetaCyc pathway level. After correcting for batch effect among different cohorts (Fig. S1C and D), we identified 161 pathways with significant differences in abundance across the three groups (Table S5). The top 30 most significantly differing pathways are illustrated in Fig. 2C. Further analysis focused on pathways associated with uric acid metabolism, revealing enhanced metabolic potential in purine nucleobases degradation I, guanosine nucleotides degradation III, and adenosine nucleotides degradation II in the gout group. Conversely, guanosine ribonucleotides *de novo* biosynthesis and adenine and adenosine salvage III metabolic potential were reduced in both HUA and gout groups. To investigate the relationships between uric acid metabolic pathways and specific genera, we computed Spearman correlation coefficients between the differential genera and the differential pathways associated with uric acid metabolism. Interestingly, genera that were enriched in the HUA and gout groups, including *Fusobacterium* and *Bilophila*, revealed positive correlations with uric acid metabolism pathways (such as the superpathway of purine nucleotide *de novo* biosynthesis I and the superpathway of guanosine nucleotides *de novo* biosynthesis I). In contrast, the *Christensenellaceae* R-7 group, which was enriched in the HC group, exhibited negative correlations (Fig. 2E). These associations were further validated through the genus-pathway correspondences identified in the PICRUSt2 analysis (Fig. 2F). These findings identify specific microbial biomarkers and metabolic pathways associated with uric acid levels, offering potential targets for improved treatment strategies.

### Network analysis revealed distinct interactions among microbial biomarkers in the HC, HUA, and gout groups

**Fig 3:**
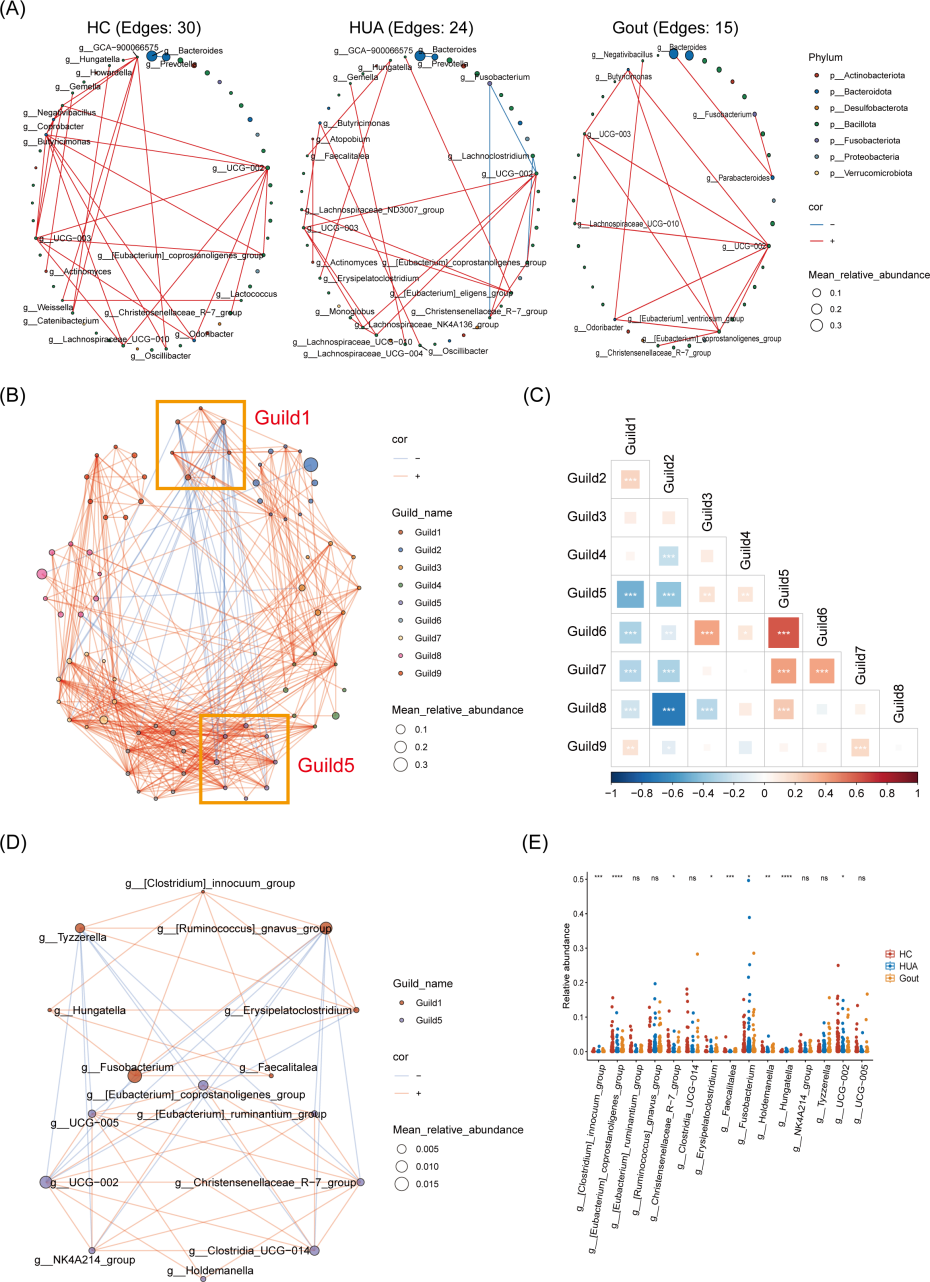
Network analysis of cross‐cohort microbial biomarkers. (**A**) Co‐occurrence bacterial networks at the genus level for each HC, HUA, and gout group. Only significantly different genera among the three groups (presented in Table S6) were shown. Node properties: (i) circle size, proportional to the mean bacterial relative abundances; (ii) colors, represent different phyla levels of genera. Edge properties: red for positive correlation and blue for negative correlation. Spearman correlation, |*r*| > 0.48 and *P* < 0.05. (**B**) Interdependent functional groups (guilds) network. Bacterial genera with at least 0.1% of mean relative abundance in at least 20% of the samples were plotted. The yellow square box represents Guild1 and Guild5 with the highest number of negatively correlated edges. Node properties: (i) circle size, proportional to the mean bacterial relative abundances; (ii) colors, represent different guilds. Edge properties: red for positive correlation and blue for negative correlation. Spearman correlation, |*r*| > 0.29 and *P* < 0.05. (**C**) Spearman correlation among different guilds. The size of the square represents |*r*|, red: positive correlation (*r* > 0); blue: negative correlation (*r* < 0). *P < 0.05, **P < 0.01, and ***P < 0.001. (**D**) Bacterial interaction network within Guild1 and Guild5. (**E**) The comparison of the relative abundance of the genus (presented in panel D) among the three groups. Kruskal-Wallis test, *P < 0.05, **P < 0.01, ***P < 0.001, ****P < 0.0001, and ns for not significant.

To explore the potential relationships among bacteria within gut microbial communities, we constructed co-occurrence networks of genera for each group using Spearman correlation with a threshold of *r* > 0.48 and *P* < 0.05 (Fig. S3A). The networks revealed distinct potential interactions among the gut microbiota of the HC, HUA, and gout groups. Specifically, the HUA and gout groups had fewer microbial connections compared to the HC group, with the HUA group exhibiting a higher number of negative correlations and reduced positive correlations (Fig. S3B), along with decreased closeness centrality (Fig. S3C). Further analysis of microbial biomarkers across the co-occurrence networks mirrored the overall microbial network findings. The HC group had more connections among key microbial biomarkers (30 edges), while the HUA and gout groups had fewer (24 and 15 edges, respectively; Fig. 3A; Table S6). These findings suggest that elevated uric acid levels might intensify microbial competition, diminishing cooperative interactions and leading to a more fragile network susceptible to disruption.

Given that bacteria in the gut ecosystem function as interdependent groups known as guilds (29), the coordinated changes within these guilds could be crucial for identifying bacteria that significantly influence uric acid levels. We constructed a co-abundance network at the genus level, identifying nine guilds with a correlation coefficient greater than 0.29 (Fig. 3B; Table S7). Among them, the Guilds with a relatively high abundance of Guild2 and Guild8. Specifically, Guild2 was more abundant in the HUA group, primarily due to *Bacteroides* (Fig. S3E), while Guild8 showed higher abundance in the gout group, primarily due to *Prevotella* (Fig. S3F). Additionally, Guild1 and Guild5 displayed a substantial negative correlation (Fig. 3C), suggesting potential antagonistic interactions within the microbial functional network. The abundance of Guild1 was notably higher in the gout group, whereas Guild5 was found to be less abundant in the HUA group (Fig. S3D). Guild1 was characterized by the inclusion of *Fusobacterium*, which was particularly enriched in both the HUA and gout groups, while Guild5 comprised *Christensenellaceae* R-7 group, observed to be less abundant in these patient groups (Fig. 3D and E). Within Guild1, *Fusobacterium* was positively correlated with *[Clostridium]_innocuum_*group and *[Ruminococcus]_gnavus_*group. In Guild5, *Christensenellaceae_*R−7_group was positively correlated with *[Eubacterium]_ruminantium_*group and *Holdemanella*. This distribution pattern implies that *Fusobacterium* and *Christensenellaceae* R-7 group may exert critical influences within their respective guilds, potentially contributing to the contrasting dynamics of the gut microbiota in relation to uric acid metabolism.

### Microbial biomarker-based classifiers effectively distinguish the HC, HUA, and gout groups

**Fig 4:**
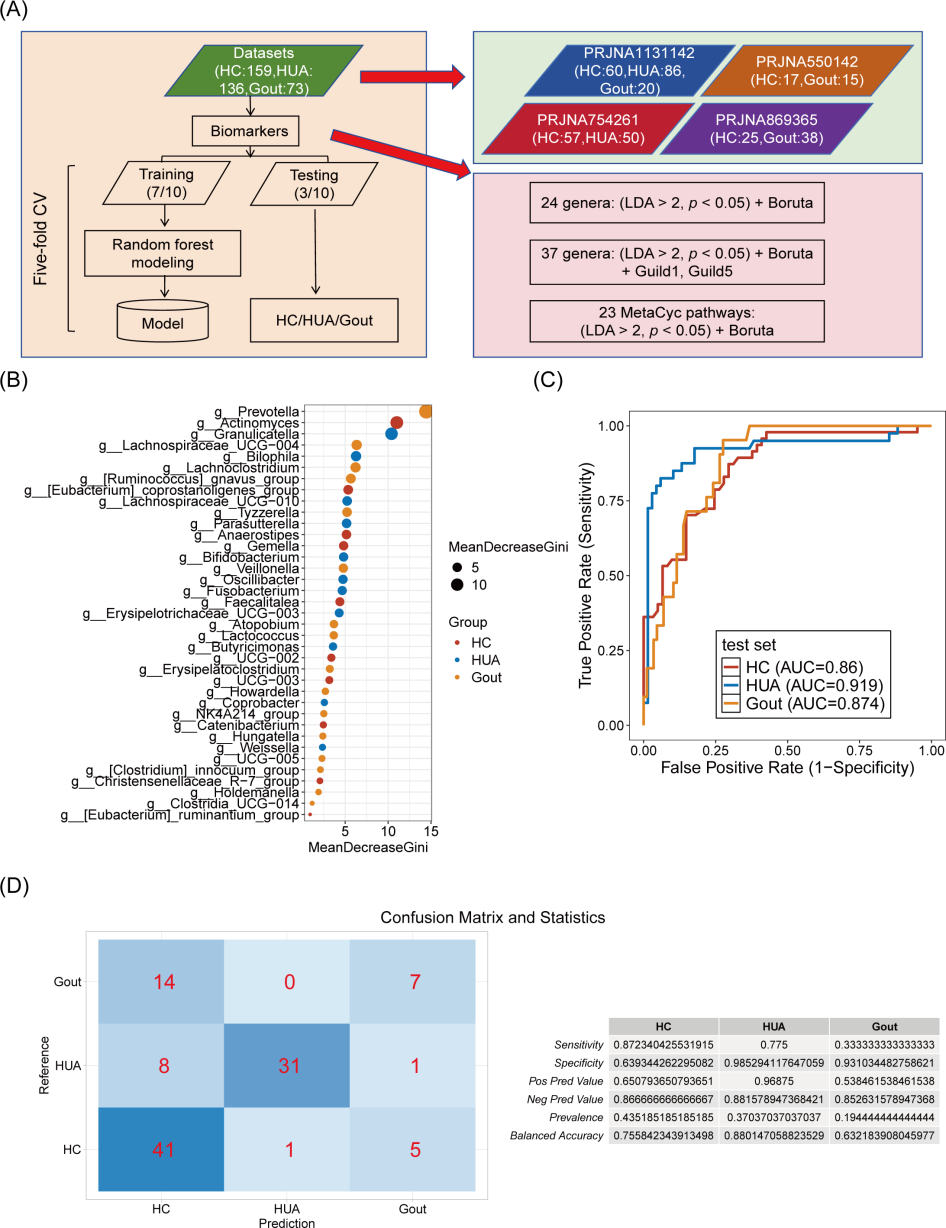
A classifier based on cross-cohort microbial biomarkers to distinguish HC, HUA, and gout individuals. (**A**) Flowchart of the classifier design procedure. (**B**) The microbial biomarkers (37 genera: [LDA > 2, *P* < 0.05] + Boruta + Guild1, Guild5) were ranked in order of importance for predicting three groups. (**C**) Receiver operating curve (ROC) for the three groups based on the microbial biomarkers shown in panel B, using the R package randomForest. The AUC for each group was indicated in parentheses; the closer the AUC to 1, the better the model performance. (**D**) The confusion matrix of the classifier constructed in panel C and its prediction performance statistics.

The distinct microbial and functional profiles observed among the HC, HUA, and gout groups prompted us to investigate whether the gut microbiota and its associated functions could serve as discriminatory factors. Utilizing a 7:3 ratio, we randomly divided 368 individuals into a training set and a test set. We then applied three distinct strategies for biomarker selection for our predictive model (see Materials and Methods; Fig. 4A). Compared with using MetaCyc pathways as biomarkers, genus as biomarkers showed higher prediction performance (Fig. S4), especially after adding Guild1 and Guild5 bacteria (Fig. 4B). The model achieved the highest prediction accuracy in the test data set, with the area under the curve (AUC) score of 0.86 for HC, 0.919 for HUA, and 0.874 for gout (Fig. 4C). To further evaluate the model’s performance, we constructed a confusion matrix, from which we extracted the model’s sensitivity, specificity, positive predictive value, negative predictive value, prevalence, and balanced accuracy (Fig. 4D). The HUA group exhibited the highest AUC of 0.919, along with the highest specificity of 0.985 and a balanced accuracy of 0.88, outperforming the HC and gout groups (Fig. 4C and D). These findings indicate that the microbial biomarker-based model possesses substantial diagnostic capability, particularly for the prediction of HUA.

### Metagenomic analysis reveals SGBs with uric acid gene clusters

Our analysis of 16S rRNA sequencing data sets revealed distinctions at the genus and MetaCyc pathway levels among HC, HUA, and gout groups. However, these data sets did not provide sufficient resolution to clarify the mechanisms by which microbes at the species level regulate uric acid levels. To address this, we analyzed Chinese metagenomic sequencing data, including 86 HC and 102 gout patients (Fig. 1A; Table S1), aiming to delve deeper into the species and their functions related to uric acid metabolism.

**Fig 5:**
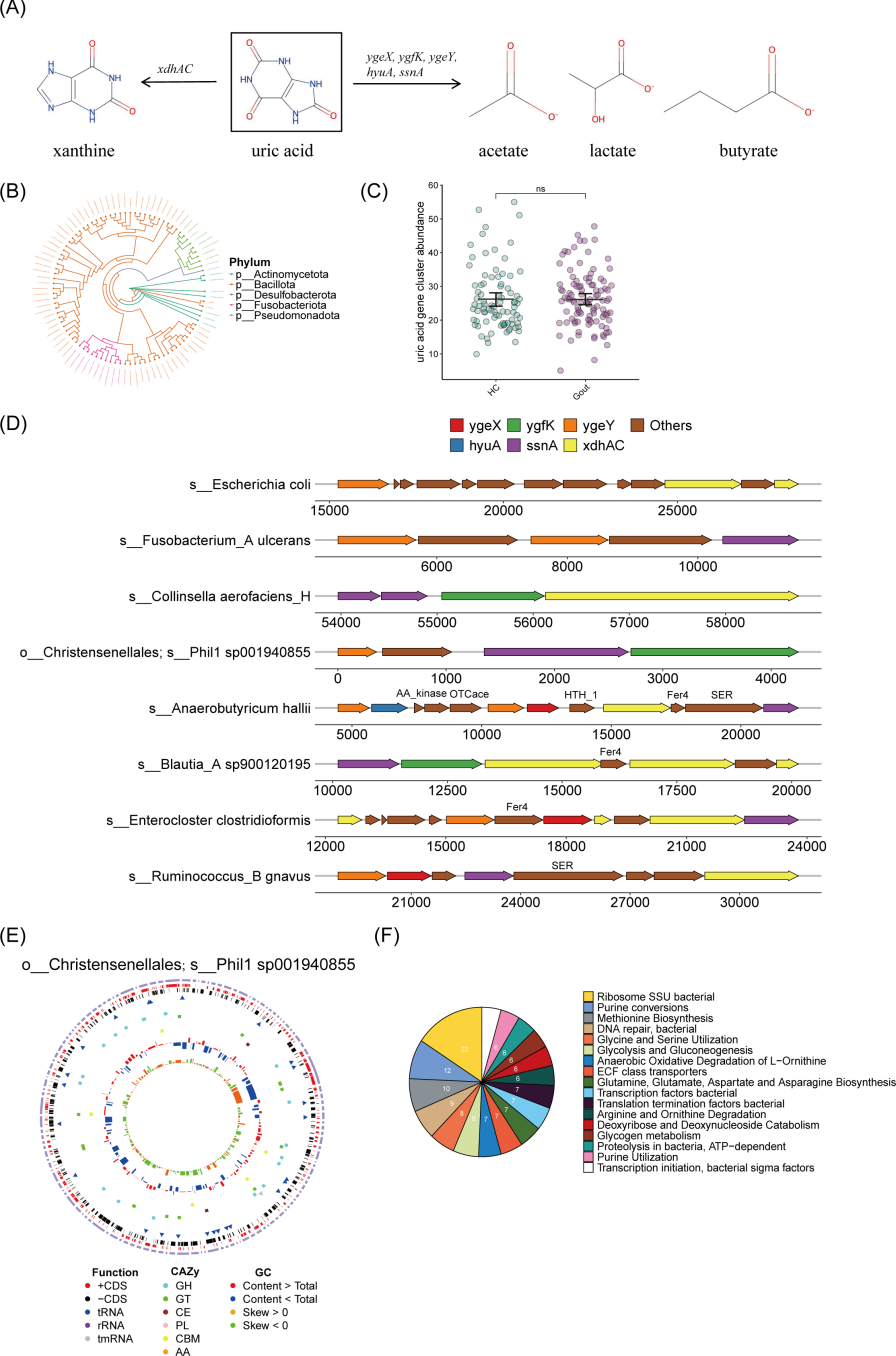
SGBs with uric acid gene cluster. (**A**) Uric acid is converted either to xanthine or lactate, and the SCFAs acetate and butyrate. (**B**) Phylogenetic tree for SGBs with the uric acid gene cluster. Clades are colored by phylum. (**C**) Comparison of the abundance of the uric acid gene cluster between the HC and gout group. Metagenomic data from study subjects were mapped to uric acid gene cluster families using gutSMASH and BiG-MAP. Resulting reads per kilobase per million reads mapped (RPKM) were normalized by cumulative sum scaling. Wilcoxon rank-sum test; ns for not significant. (**D**) Genomic context for uric acid metabolic genes from representative SGBs with the uric acid gene cluster. (**E**) Genomic features of *o__Christensenellales; s__Phil1 sp001940855*. The 1,966,753 bp genome, containing 128 contigs with an N50 length of 20,111 bp and a GC content of 53%, encodes 1,890 genes. These are predominantly protein-coding (1,847 genes, or 97.7%), with a minority being RNA-coding genes (43 genes, or 2.3%). From the outer circle to the inner, it represents the length of contigs, coding sequences (CDS) on forward and reverse strands, tRNA, rRNA, tmRNA, carbohydrate-active enzymes (CAZy) annotation, GC content, and GC skew curve, respectively. (**F**) Pie chart showing the function of *Phil1 sp001940855* genome based on Rapid Annotations using Subsystems Technology (RAST) annotation.

Previous research has reported that a conserved set of uric-acid-inducible genes, including *ygeX*, *ygfK*, *ygeY*, *hyuA*, *ssnA*, and *xdhAC*, is widely distributed across diverse bacterial taxa (encompassing four phyla: Actinobacteria, Bacillota, Fusobacteria, and Proteobacteria). These genes participate in the anaerobic consumption of uric acid, converting it into xanthine or lactate, as well as the short-chain fatty acids (SCFAs) acetate and butyrate (Fig. 5A) (18). By analyzing the metagenome data set, we discovered 94 SGBs containing the uric acid gene cluster (Table S9). Phylogenetic analysis indicated that these genes are widely distributed across SGBs from five different phyla, with Bacillota phylum showing the highest prevalence of SGBs with these gene clusters (Fig. 5B). However, we did not observe a significant difference in the abundance of the uric acid gene cluster between the HC and gout groups (Fig. 5C). Consistent with prior studies (18, 30), we found that uric acid metabolic genes are conserved in gene clusters within diverse SGBs, including *Anaerobutyricum hallii*, *Blautia_*A sp900120195, *Collinsella aerofaciens*, *Enterocloster clostridioformis*, and *Ruminococcus_*B gnavus. Among them, the first three species not only contain the uric acid metabolism gene, but also share *Fer4*, which plays the function of iron storage (Fig. 5D). Of particular interest, our study identified for the first time an SGB carrying a cluster of uric acid metabolism genes (including *ygeY*, *ssnA*, and *ygfK*) in *Christensenellales*, named *Phil1 sp001940855* (26, 31). To further investigate, we reassembled the genome of *Phil1 sp001940855*, which is nearly complete (96.45%), with minimal contamination (0.81%). The genome comprises a single circular chromosome with a size of 1,966,753 bp (GC content, 53%) (Fig. 5E). Functional analysis showed that *Phil1 sp001940855* is enriched in pathways related to ribosome SSU bacterial, purine conversions, and methionine biosynthesis (Fig. 5F). The purine conversions pathway includes adenine phosphoribosyltransferase (APRT) and hypoxanthine-guanine phosphoribosyltransferase (HGPRT), which are involved in purine recycling and may reduce uric acid production. Additionally, enzymes associated with purine utilization pathways were also found in *Phil1 sp001940855* (Fig. S5). In summary, our metagenomic analysis indicated that SGBs encoding uric acid genes are widely present in the human gut microbiota and may regulate uric acid levels through enzymes associated with purine metabolism.

## DISCUSSION

Extensive research has highlighted the complex interplay between the gut microbiota and HUA and gout, sparking considerable interest in the potential of the gut microbiota to regulate uric acid metabolism. However, previous studies predominantly focused on single cohorts and did not delve into the specific functionalities of bacteria in uric acid metabolism, which limited the broad applicability of their findings. To overcome these limitations, our study conducted a comprehensive multi-cohort analysis to evaluate the gut microbiota structure, specific microbial classification, and functional characteristics in the HC, HUA, and gout groups. We established a diagnostic model based on gut microbial biomarkers and identified species with uric acid metabolic potential, offering a new perspective on the relationship between the gut microbiota and HUA and gout, and laying a theoretical foundation for the development of interventions to modulate uric acid levels through the regulation of the gut microbiota.

Consistent with previous research (11), our study observed a significant reduction in microbial richness in the gout group, alongside notable differences in microbial community structure among the three groups. However, contrary to the findings reported by Sheng et al. (10), we did not detect a decrease in microbial diversity indices in the HUA group. This discrepancy may suggest that the inflammatory state associated with gout exacerbates the impact on microbial diversity more than HUA. Further analysis of the genera with significantly altered abundance in HUA and gout groups revealed a marked reduction in *Christensenellaceae* R-7 group, *Anaerostipes,* and *Collinsella*. Bacteria within the *Christensenellaceae* family are implicated in nutrient absorption and modulation of intestinal immune function (26, 32). Notably, *Christensenellales* has been implicated in modulating glucose and lipid metabolism (26, 31) and is linked to an increased risk of atherosclerotic cardiovascular disease (33). Given that serum uric acid levels and purine metabolites are positively correlated with atherosclerotic cardiovascular disease (30, 34), these findings collectively suggest that *Christensenellales* may play a role in regulating uric acid metabolism. *Anaerostipes* and *Collinsella*, known for their ability to produce butyrate, could mitigate inflammatory responses (35, 36), suggesting a possible role in alleviating inflammation associated with gouty flares. Pro-inflammatory bacteria such as *Fusobacterium* and *Bilophila* were enriched in both HUA and gout groups, potentially facilitating the accumulation of uric acid crystals (37, 38). Moreover, while *Lactobacillus* and *Lactococcus* are generally recognized for their beneficial roles in promoting nutrient absorption and preventing intestinal diseases (39), excessive lactate production in the gout group might competitively affect renal tubular secretion of uric acid, lowering blood pH and consequently diminishing the solubility of uric acid (40). This could lead to the deposition of uric acid crystals and a reduction in uric acid excretion. Our results indicated that targeted modulation of specific bacteria can effectively stabilize serum uric acid levels. The potential therapeutic applications of these findings warrant further investigation, particularly in the context of developing dietary or microbial interventions for HUA and gout.

The gut microbiota plays a pivotal role in modulating host metabolism and immune function (41). Our study revealed significant alterations in pathways related to uric acid metabolism in both HUA and gout groups. Notably, in the gout group, we observed enrichment of degradation pathways for purine bases, guanosine nucleotides, and adenosine nucleotides, which may reflect an accelerated rate of purine metabolism and consequently elevated uric acid production during gout flares. Conversely, in the HUA and gout groups, we observed reduced abundance in the *de novo* biosynthesis of guanosine ribonucleotides and the salvage pathways for adenine and adenosine. The salvage of adenine and guanine primarily depends on phosphoribosyltransferases, such as APRT and HGPRT. When the abundance of these salvage pathways is reduced, free purine bases (adenine and guanine) cannot be efficiently recycled for nucleotide synthesis. Instead, they are shunted into degradation pathways and ultimately oxidized to uric acid (42). The beneficial bacteria *Christensenellaceae* R-7 group showed a negative correlation with uric acid metabolic pathways, while pro-inflammatory bacteria such as *Fusobacterium* and *Bilophila* demonstrated a positive correlation with uric acid metabolic pathways, further substantiating the role of specific bacteria in regulating uric acid metabolism. However, it should be noted that the metabolic pathway changes identified in our analysis were merely predicted based on 16S rRNA data. A variety of factors, such as regulation, cellular transport, and kinetics, can influence bacterial metabolic capacity. Future research should integrate multi-omics approaches (e.g., metagenomics, metabolomics, and proteomics) to verify the mechanistic role of the gut microbiota in regulating uric acid metabolism.

The interplay between the gut microbiota and host health is well-established, with the microbiota’s dynamics reflecting and influencing the host’s physiological state (43). The abundance of keystone species is a critical driver maintaining the complexity and stability of microbial community networks (44). In the co-occurrence networks of HUA and gout groups, a reduction in interactions among these key species may adversely affect the functionality of the gut microbiota, impacting the host’s physiological status. Beyond the co-occurrence networks that reveal mere correlations, an analysis based on functional guilds captures synergistic microbial communities. Previous studies have identified two competing guilds within the gut microbiota in metabolic diseases such as obesity and diabetes, exerting opposing functions during disease progression (29, 45). Similarly, our study discovered two guilds with significant negative correlations in the gut microbiota of HUA and gout patients: one containing pro-inflammatory bacteria *Fusobacterium* and the other beneficial bacteria *Christensenellaceae* R-7 group, indicating their contrasting roles in the microbial functional network and their impact on host uric acid metabolism levels. Intriguingly, the inclusion of bacteria from these guilds in our predictive model enhanced its performance, underscoring their significance. While a prior study has identified reduced *Christensenellaceae* abundance and increased *Fusobacterium* prevalence in HUA (46), no predictive models leveraging these microbial signatures have been developed. Existing classifiers (11, 19, 21, 47) exhibit moderate discriminative power (mean AUC ≈ 0.80), yet face three key limitations: (i) reliance on single-cohort analyses, (ii) identification of microbial markers solely through differential abundance analysis, and (iii) construction as dichotomous models (e.g., HC vs HUA or HC vs gout), limiting their generalizability. To address these shortcomings, we developed a multi-class random forest classifier. This model incorporates multi-cohort data and integrates microbial markers selected not only by differential abundance but also by considering species within functional guilds exhibiting significant synergistic interactions. Consequently, our approach simultaneously discriminates among HC, HUA, and gout subjects with enhanced robustness (AUC > 0.85) and greater clinical utility. However, the predictive capacity at the functional pathway level was relatively low, potentially due to the inaccuracy of function prediction from 16S rRNA sequencing data. Future research should incorporate more metagenomic data to conduct functional studies, providing a more comprehensive understanding of the gut microbiota’s role in uric acid metabolism.

**Fig 6:**
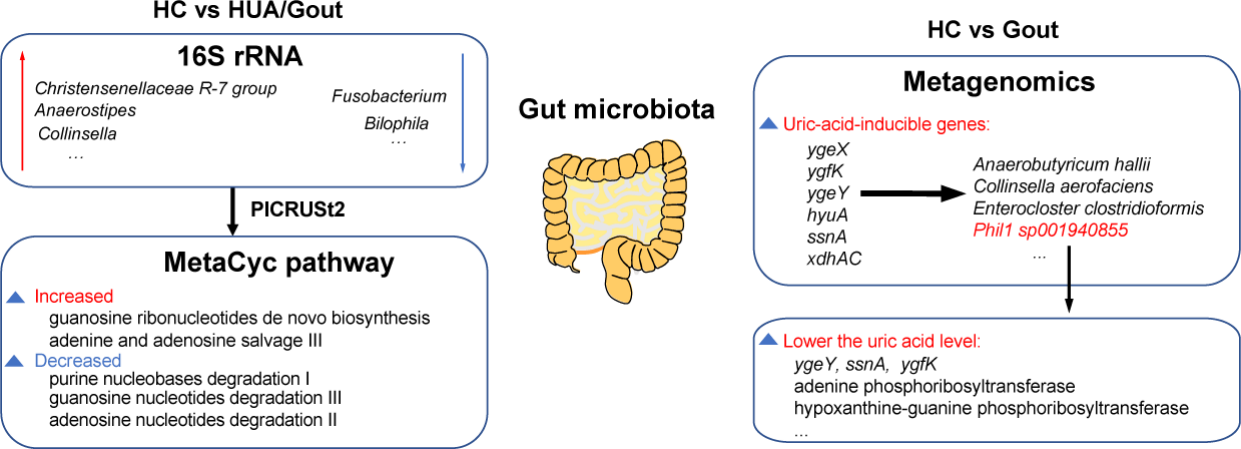
An overview diagram illustrating the link between the gut microbiome and HUA and gout.

Recent studies have reported that bacteria carrying gene clusters for uric acid metabolism are broadly capable of degrading uric acid (18, 30). In alignment with these findings, our investigation identified several species known to carry these gene clusters, such as *Anaerobutyricum hallii*, *Collinsella aerofaciens*, and *Enterocloster clostridioformis*. Notably, *Anaerobutyricum hallii* and *Collinsella aerofaciens*, which are recognized for their ability to produce butyrate and exert anti-inflammatory effects (25, 48), were found to be less abundant at the genus level in both HUA and gout groups. This observation suggests a mechanistic link between their abundance and uric acid metabolism. Furthermore, prior genomic studies of uric acid-metabolizing bacteria did not report *Christensenellales* strains harboring this gene cluster (18, 30). Notably, we identified a *Christensenellales* SGB (*Phil1 sp001940855*, GTDB annotation) assembled from metagenomic data. This SGB was identified for the first time as carrying a uric acid metabolism gene cluster, including *ygeY*, *ssnA*, and *ygfK*, suggesting its potential to degrade uric acid into SCFAs. Furthermore, the SGB encodes the key enzymes involved in the purine recycling pathway, particularly APRT and HGPRT. The dysfunction of these enzymes can lead to an increase in purine nucleotide degradation, thereby causing an increase in uric acid levels (Fig. 6) (42). This discovery provides a new target for precision medicine strategies aimed at regulating uric acid levels through the gut microbiota. However, no significant difference was observed in the abundance of the uric acid metabolic gene cluster between the HC and gout groups, indicating the need for further exploration of novel gene clusters with uric acid metabolic functions. In addition, we noticed that although uric acid metabolism genes are widely distributed in intestinal bacteria, the gene combinations and orders carried by different bacteria are different, which may lead to certain differences in their uric acid metabolism capabilities. For instance, some bacteria can completely break down uric acid into metabolites such as xanthine and short-chain fatty acids, while some bacteria can only partially metabolize uric acid into short-chain fatty acids.

Our study presents several strengths, including the integration of multiple cohorts, the application of a unified analysis method, and the identification of species involved in uric acid metabolism. However, due to limited data access and incomplete sample information, we were unable to control for confounding factors such as diet, age, and gender, which may influence the gut microbiota composition and its metabolic functions. Additionally, our research was confined to cohorts from China, underscoring the necessity for multicenter studies across diverse regions and countries to validate the universality of our predictive model. Future studies should integrate multi-omics approaches with controlled interventions to clarify the causal relationships by which specific gut microbes modulate uric acid metabolism, providing a more comprehensive understanding of the complex interplay between the gut microbiota and host metabolism.

### Conclusions

In summary, this comprehensive multi-cohort study deepens our understanding of the relationship between the gut microbiota and the development of HUA and gout. An effective prediction model based on microbial biomarkers was successfully established to distinguish HC, HUA, and gout individuals. Most notably, *Phil1 sp001940855* was discovered for the first time to carry a uric acid metabolic gene cluster and possess enzymes related to purine metabolism, providing a new target for precision medical interventions. As our understanding of the gut microbiota continues to expand, we anticipate the development of innovative therapeutic approaches to meet the treatment needs of patients with HUA and gout.

## MATERIALS AND METHODS

### Data collection

We searched the gut microbiome data sets associated with high uric acid in the PubMed and NCBI BioProject databases using the following search terms: (“hyperuricemia” OR “gout” OR “uric acid”) AND (“gut microbiome” OR “gut microbiota” OR “human microbiome” OR “Intestinal microbiota”).

Our selection criteria were as follows:

- Human fecal samples were the basis of the study.
- Clear disease categorization was provided, with no fewer than six valid samples in both the case and control groups.
- Public availability of raw sequences, barcodes, and metadata, along with a sequencing depth exceeding 3,000 reads.

After eliminating duplicates and data sets that lacked comprehensive metadata or did not meet the minimum sample requirements, we identified and selected a total of five data sets. Among these data sets, PRJNA1131142, PRJNA754261, PRJNA869365, and PRJNA550142 consist of 16S rRNA sequencing data, while the data set CNP0000284 comprises metagenomic next-generation sequencing data (Table S1).

### Raw 16s rRNA sequencing data processing

Raw sequencing data were obtained from the NCBI Sequence Read Archive database. Bioinformatics analysis of the microbiome was conducted using QIIME2 v2023.5.1 (49). The process began with demultiplexing and quality filtering of the raw sequences utilizing the q2-demux plugin, followed by sequence denoising with the DADA2 algorithm. Taxonomic classification of amplicon sequence variants was performed with the q2-feature-classifier classify-sklearn tool, which employed a naive Bayes classifier with reference to the SILVA database release 138. Only bacterial genera that constituted at least 0.01% of the mean relative abundance in a minimum of 10% of the samples were retained for further analysis.

Alpha diversity, measured by richness and the Shannon index, was computed using the R package microbiome v1.22.0. Beta diversity, assessed through PCoA based on Bray-Curtis dissimilarity metrics, was computed using the R package vegan v2.6.4. These analyses were conducted after rarefying the samples to the minimum sequencing depth across the data set. Functional profiles of microbial communities were predicted using Phylogenetic Investigation of Communities by Reconstruction of Unobserved States (PICRUSt2) v2.5.2 (50) based on the Metabolic Pathways From all Domains of Life (MetaCyc) database. MetaCyc pathways related to uric acid metabolism were identified and selected using the keywords “adenine, guanine, xanthine, purine, nucleotide, adenosine, guanosine, and inosine.”

### Batch effects removal for microbiome data across cohorts

To address batch effects in microbiome data across different cohorts, we employed the R package ConQuR v2.0. Batch effects can complicate comprehensive analysis and introduce false positives, potentially hindering the development of predictive models and biomarkers. This software adjusts for these effects by treating each item ID as a distinct batch. ConQuR is a robust method that accommodates the complex distributions of microbial read counts by non-parametric modeling, generating batch-removed zero-inflated read counts that can be used in and benefit from usual subsequent analyses (51). In addition, we performed PCoA to graphically represent the impact of batch effects on the structure of microbial communities based on Bray-Curtis dissimilarity metrics derived from microbiome data. The extent of batch effects was quantified using the *R*-value and *P*-value from the ANOSIM test. A *P*-value exceeding 0.05, in conjunction with a minimal R-value, indicates a reduced contribution of batch effects in the model, diminishing their influence on model construction. After removing batch effects, we proceeded with further modeling and biostatistical analysis of the relative abundance data from the integrated cohorts, ensuring a more accurate and reliable interpretation of the microbiome data in the context of our study.

### Biomarker identification using linear discriminant analysis effect size

We used the R package microeco v1.5.0 to conduct LEfSe analysis (52), aimed at identifying taxa and pathways that are specific to the disease states. The LEfSe analysis output provides effect size scores, represented as the linear discriminant analysis (LDA) scores, which reflect the degree of difference among HC, HUA, and gout groups. The higher the score, the more significant the difference. Taxa and pathways with *P* < 0.05 and LDA score >2 were initially identified as potential biomarkers.

### Microbial network analysis

To elucidate the relationships among various bacterial genera, we constructed a co-occurrence network based on the 16S rRNA sequencing data. Spearman correlation was applied to define the associations between bacteria in the HC, HUA, and gout groups, respectively. Only genera with a Benjamini–Hochberg-corrected *P*-value of less than 0.05 in each group and present in at least 20% of the samples were retained. The final network construction was achieved using random matrix theory (RMT) with the R package RMThreshold v1.1. This method identifies *r*-value thresholds of correlation coefficients based on the transition of the empirical nearest-neighbor eigenvalue spacing distribution from the Gaussian orthogonal ensemble to a Poisson distribution, indicating the point at which the inherent structure of the network is separated from noise (53).

Additionally, bacterial species within the human gut exist as interdependent functional groups, known as guilds, which adapt and respond to environmental changes (29). Co-abundance analysis was conducted to identify these guilds. Spearman correlation was again utilized to calculate the relationships between the relative abundances of different genera. These correlation values were transformed into correlation distances (1 − correlation value), and the genera were subsequently clustered using the Ward clustering method. Guild identification was performed by segmenting the cluster tree with permutational multivariate analysis of variance, employing 9,999 permutations and a significance level of *P* < 0.001. This process was facilitated by the adonis function within the R package vegan v2.6.4. For network visualization and to assess the centrality of each node, we employed the R package ggClusterNet v0.1.0. This allowed us to graphically represent the network and quantify the relative importance of each bacterial genus within the interconnected microbial ecosystem.

### Random forest classifier model

To determine if microbial-related biomarkers could distinguish HC from the HUA and gout groups, we used the R package randomForest v4.6.14 to construct a classification model for disease diagnosis. Biomarkers, including genera and MetaCyc pathways identified by LEfSe analysis with a *P*-value below 0.05 and an LDA score above 2, were initially selected. We then applied the Boruta feature selection method via the R package Boruta v8.0.0 to refine the optimal subset of features. Additionally, we incorporated genera from the key guilds Guild1 and Guild5 as supplementary biomarkers. Finally, three microbial marker strategies were selected:

- 24 genera: selected based on LDA score >2 and *P* < 0.05, followed by Boruta feature selection.
- 37 genera: the above 24 genera and genera from Guild1 and Guild5.
- 23 MetaCyc pathways: selected based on LDA score >2 and *P* < 0.05, followed by Boruta feature selection.

To prevent overfitting, the data set was randomly split into training and testing sets in a 7:3 ratio. Models were trained using the training set and validated using the test set. During training, we employed fivefold cross-validation with the R package caret v6.0.88. The performance of the random forest classification models was evaluated using the receiver operating characteristic (ROC) curve analysis, facilitated by the R package pROC v1.17.0.1. The AUC was utilized to measure predictive accuracy, with AUC values closer to 1 indicating superior model performance. The Gini importance scores of the selected biomarkers were determined by setting the “importance = TRUE” parameter in the R package randomForest v4.6.14. To assess the model’s predictions against actual outcomes, we utilized a confusion matrix, which illustrates the instances within each actual class as rows and those within each predicted class as columns. By examining the model’s sensitivity, specificity, positive predictive value, negative predictive value, prevalence rate, and balanced accuracy, we gained further insights into its classification capabilities and its propensity for prediction errors.

### Raw metagenomic sequencing data processing

Raw sequence reads were preprocessed for quality and adapter trimming using Trimmomatic v0.33, ensuring the removal of low-quality regions. Subsequently, contaminating human reads were eliminated with Bowtie2 v2.4.1, referencing the GRCh38 reference database. Following the exclusion of host reads, individual samples' sequences were assembled using Megahit v1.2.9, integrated within MetaWRAP v1.3.2 (54). The assembled metagenomic data were then binned to derive draft metagenome-assembled genomes (MAGs), employing tools such as CONCOCT v1.0.0, MaxBin2 v2.2.6, and metaBAT2 v2.12.1. The quality of the MAGs was evaluated using CheckM v1.0.12, which estimated genome completeness and contamination levels. MAGs with a completeness score above 70% and a contamination level below 10% were selected for further analysis. High-quality MAGs were clustered using dRep v3.4.3 to discern the most representative genomes, resulting in a curated list of 609 SGBs (Table S8). Taxonomic classification for each SGB was predicted using GTDB-Tk v2.3.2 (55), while PROKKA v1.14.6 was utilized to identify the location of coding genes within each SGB. DIAMOND v2.18 was then used to search all predicted proteins against the carbohydrate-active enzymes (CAZy) database via dbCAN4 (56).

To delineate the genetic relationships among the bins, a maximum likelihood phylogenetic tree was constructed using PhyloPhlAn v3.0.67, based on concatenated protein sequence alignments. This taxonomic and phylogenetic data were integrated and visualized using ggtree v3.8.0. Finally, the functions of SGBs were predicted using the Rapid Annotations using Subsystems Technology (RAST) platform (57).

### Metagenomic analysis of uric acid gene cluster

To analyze the uric acid gene cluster within SGBs, we created a uric acid detection rule leveraging six pivotal marker genes (*ygeX*, *hyuA*, *ygfK*, *ssnA*, *ygeY,* and *xdhAC*) for the metabolic gene cluster (MGC) prediction software gutSMASH (58), as outlined in previous research (18). The resulting uric acid predicted MGCs were then used as input for the gene cluster abundance assessment tool BiG-MAP (59). The resulting reads per kilobase per million reads mapped (RPKM) counts were then normalized using cumulative sum scaling from the R package metagenomeSeq v1.42.0 to correct for differences in sequencing depth.

### Statistical analysis and bioinformatics methods

Data integration and statistical analysis were performed using R v4.3.1. Boxplots and barplots were created using the R package ggpubr v0.6.0 and ggplot2 v3.5.0. The R package VennDiagram v1.7.3 was used to generate Venn diagrams, and heatmaps were produced with the R package pheatmap v1.0.12. Spearman correlation analysis was performed using the R package corrplot v0.92. Sankey diagrams were plotted with the R package networkD3 v0.4. Wilcoxon rank sum test was used for statistical comparison between two groups, Kruskal-Wallis test was used for comparison across multiple groups, and *P*-values were adjusted with the Benjamini-Hochberg method. *P < 0.05, **P < 0.01, ***P < 0.001, ****P < 0.0001, and ns for not significant (*P* > 0.05).

## Data Availability

The 16S rRNA raw sequence data used in the current study are available in the NCBI Sequencing Read Archive under the accession numbers PRJNA1131142, PRJNA754261, PRJNA869365, and PRJNA550142. The metagenome raw sequence data used in the current study are available in the CNGB Sequence Archive with the accession number CNP0000284. The analysis code is available at https://github.com/jinlong418/Hyperuricemia-RF-model.

## SUPPLEMENTAL MATERIAL

Supplemental figures msystems.01091-25-s0001.docx Fig. S1 to S5.

Supplemental tables msystems.01091-25-s0002.xlsx Tables S1 to S9.

ASM does not own the copyrights to Supplemental Material that may be linked to, or accessed through, an article. The authors have granted ASM a non-exclusive, world-wide license to publish the Supplemental Material files. Please contact the corresponding author directly for reuse.
