# Supplementary material for: Multi-cohort analysis unveils novel microbial targets for the treatment of hyperuricemia and gout
Source: mSystems. 2025 Sep 17;10(10):e01091-25. doi: 10.1128/msystems.01091-25 (PMC12542679; doi:10.1128/msystems.01091-25)
Supplement: Supplemental figures — Fig. S1 to S5. [file msystems.01091-25-s0001.docx]

**Supplementary figures**

**
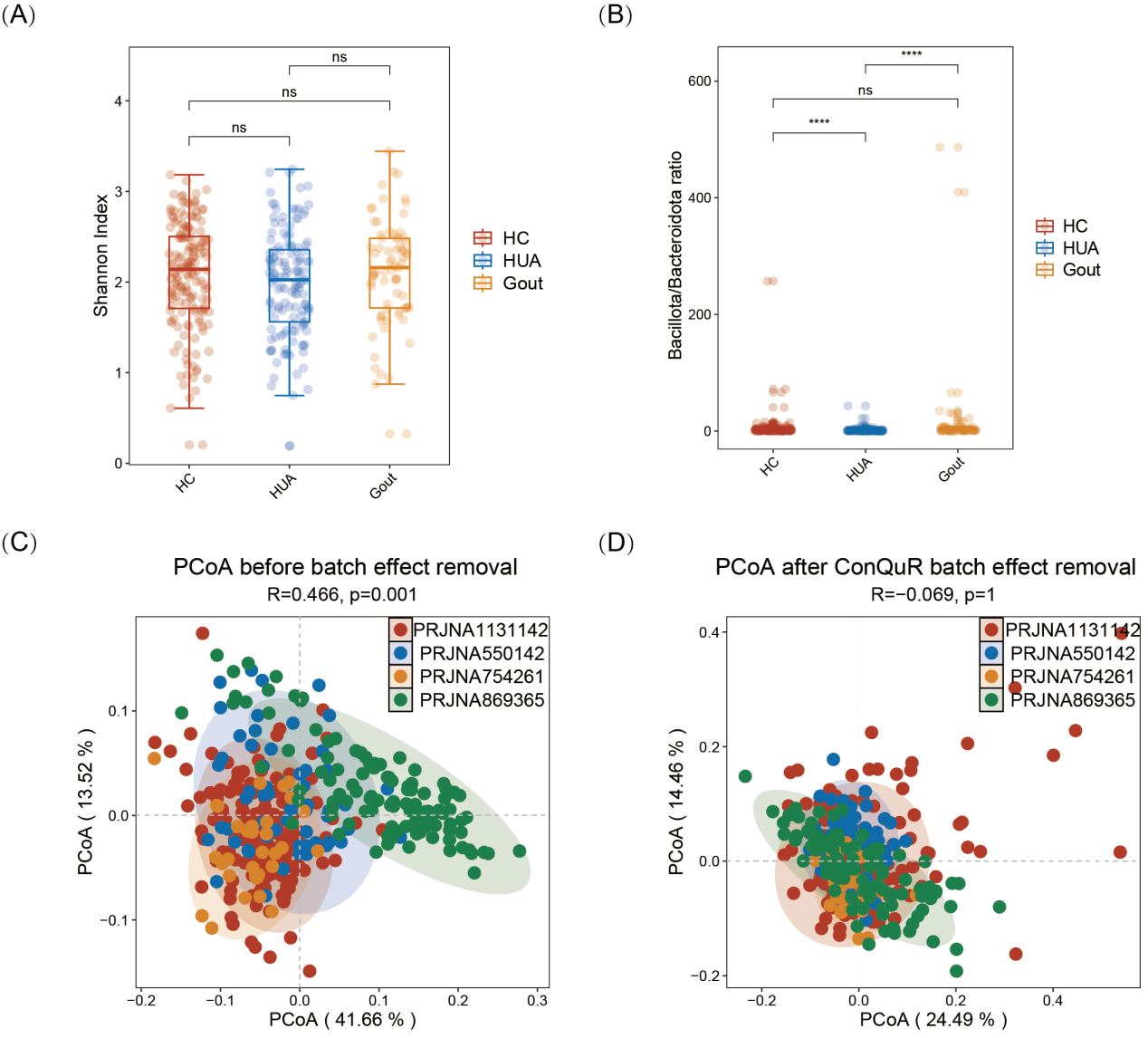
**

Figure S1. Batch effects removal for microbial functional abundance across cohorts. (A) Alpha diversity indices of genera among the three groups according to Shannon index. Significance was determined using the Wilcoxon rank-sum test. ns for not significant. (B) The Bacillota/Bacteroidetes ratio of the three groups. Wilcoxon rank-sum test. *****p* < 0.0001; ns for not significant. HC, healthy controls; HUA, patients with hyperuricemia; (C) Principal coordinates analysis (PCoA) plot shows all samples using MetaCyc pathway level Bray-Curtis dissimilarity colored by cohorts before batch-effect removal. ANOSIM, R = 0.466, p = 0.001. (D) Principal coordinates analysis (PCoA) plot shows all samples using MetaCyc pathway level Bray-Curtis dissimilarity colored by cohorts after batch-effect removal by R package ConQuR. ANOSIM, R = -0.069, p = 1.


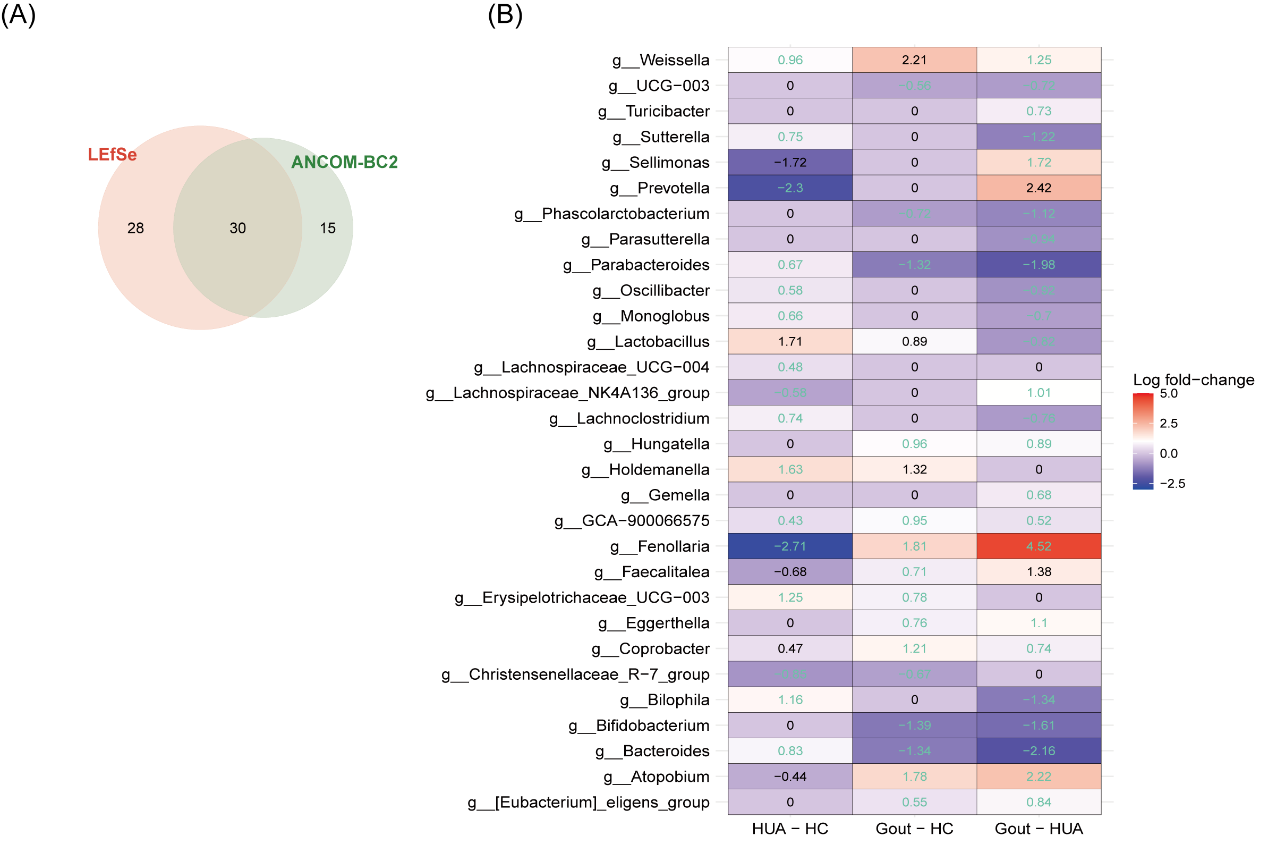


Figure S2. Differential genera among the HC, HUA and gout groups were analyzed by ANCOM-BC2. (A) Venn diagram of shared and unique genera with significant differences among the three groups using LEfSe and ANCOM-BC2. (B) Heatmap of shared genera with significant differences analyzed by LEfSe and ANCOM-BC2. The columns denote the specific comparisons: HUA versus HC, gout versus HC and gout versus HUA. The rows list significant genus as identified by ANCOM-BC2. Each cell is color-coded to represent significant changes in absolute abundance: purple represents reduced abundance and red indicates increased abundance. Multiple testing corrections were performed using the Holm–Bonferroni method. The text within each cell represents the log fold-change value. The log fold-change values displayed in black represent significant changes without adjustment for mdFDR, whereas those in green are significant after applying mdFDR control.


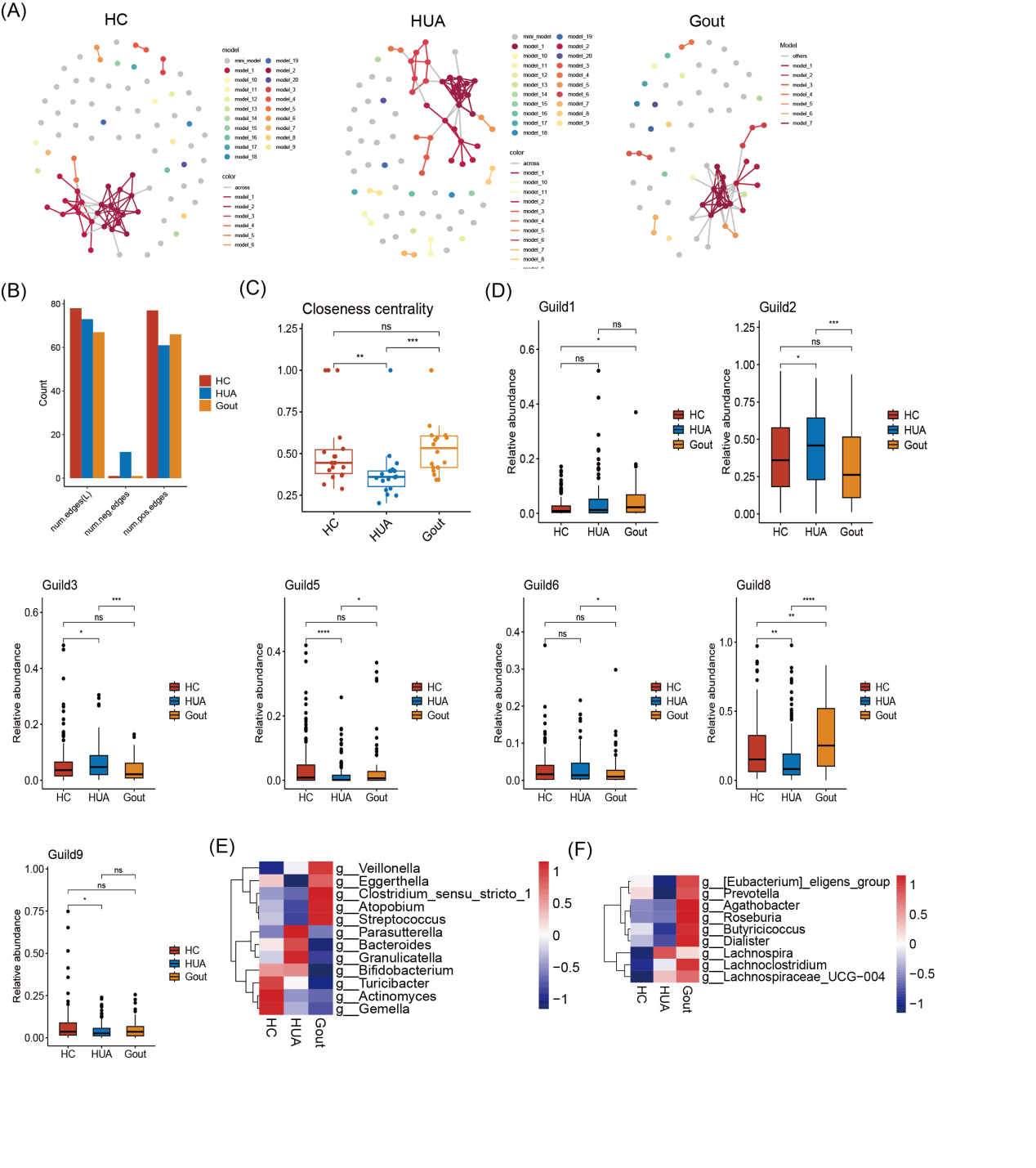


Figure S3. Microbial interactions in the gut of HC, HUA, and gout individuals. (A) Network plots describing co-occurrence of bacterial genera in the gut microbiota of the three groups based on the Spearman correlation algorithms (|r| > 0.48, *p* < 0.05). Bacterial genera with at least 0.01% of mean relative abundance in at least 20% of the samples were plotted. Each node presents a bacterial genus. The colors of the nodes represent different models. (B) The number of total, positively correlated, and negatively correlated edges in the three groups of co-occurrence networks. (C) Comparison of nodes closeness centrality in the three groups of co-occurrence networks. Wilcoxon rank-sum test. ***p* < 0.01, ****p* < 0.001 and ns for not significant. (D) Comparison of guilds relative abundance in the three groups. Only the 7 significantly different guilds among the three groups were shown. Wilcoxon rank-sum test. **p* < 0.05, ***p* < 0.01, ****p* < 0.001, *****p* < 0.0001 and ns for not significant. (E) Heatmap shows the mean relative abundance of the three groups of bacteria in Guild2. (F) Heatmap shows the mean relative abundance of the three groups of bacteria in Guild8.


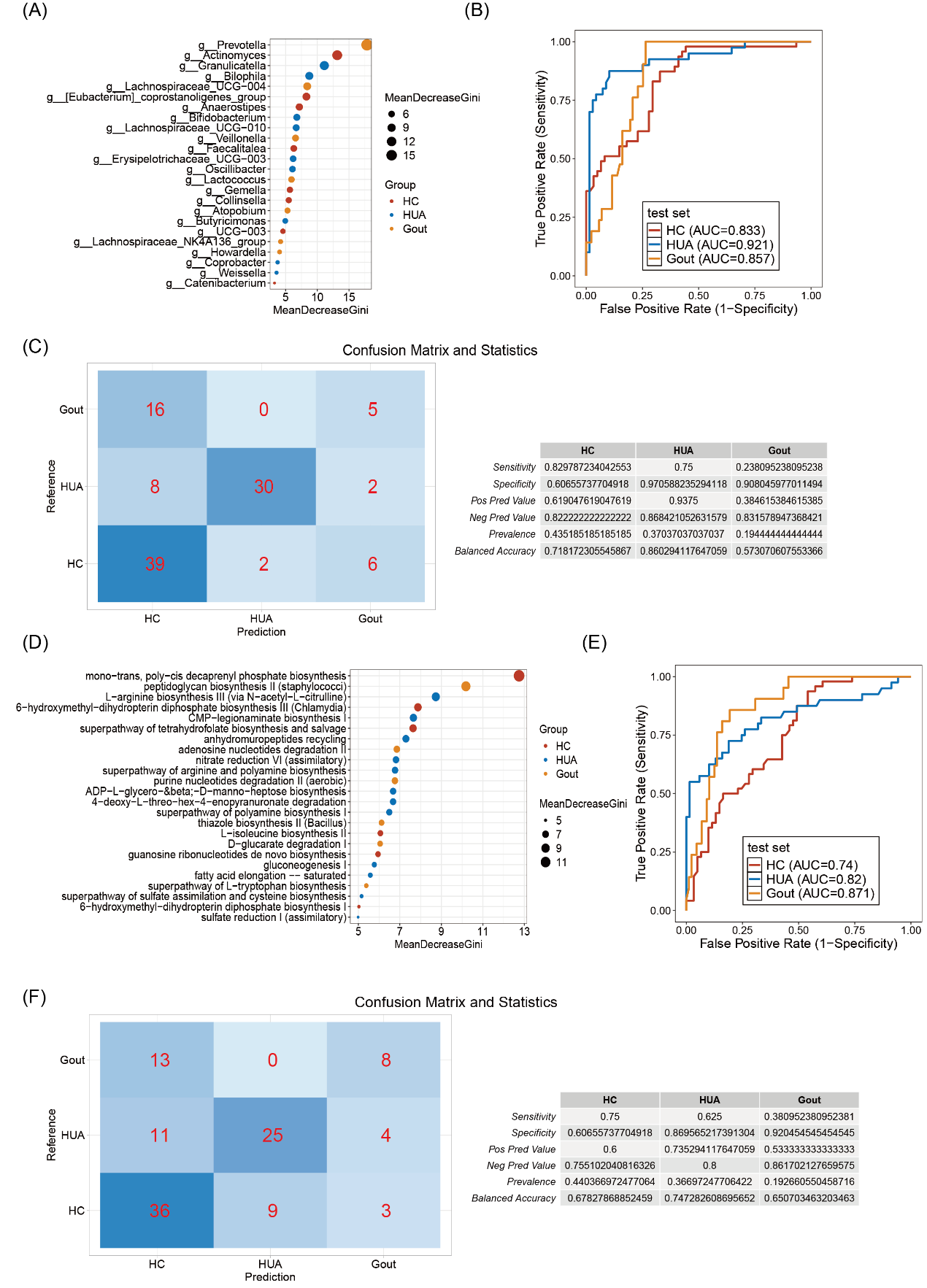


Figure S4. Classifiers based on taxonomic and functional profiles to distinguish HC, HUA, and gout individuals. (A) The biomarkers (24 genera: (LDA > 2, *p* < 0.05) + Boruta) were ranked in order of importance for predicting three groups. (B) Receiver operating curve (ROC) for the three groups based on the biomarkers shown in Figure S4A using R package randomForest. The area under the curve (AUC) for each group was indicated in parenthesis, the closer the AUC to 1, the better the model performance. (C) The confusion matrix of the classifier constructed in Figure S4A and its prediction performance statistics. (D) The biomarkers (23 MetaCyc pathways: (LDA > 2, *p* < 0.05) + Boruta) were ranked in order of importance for predicting three groups. (E) Receiver operating curve (ROC) for the three groups based on the biomarkers shown in Figure S4D using R package randomForest. The AUC for each group was indicated in parenthesis, the closer the AUC to 1, the better the model performance. (F) The confusion matrix of the classifier constructed in Figure S4D and its prediction performance statistics.


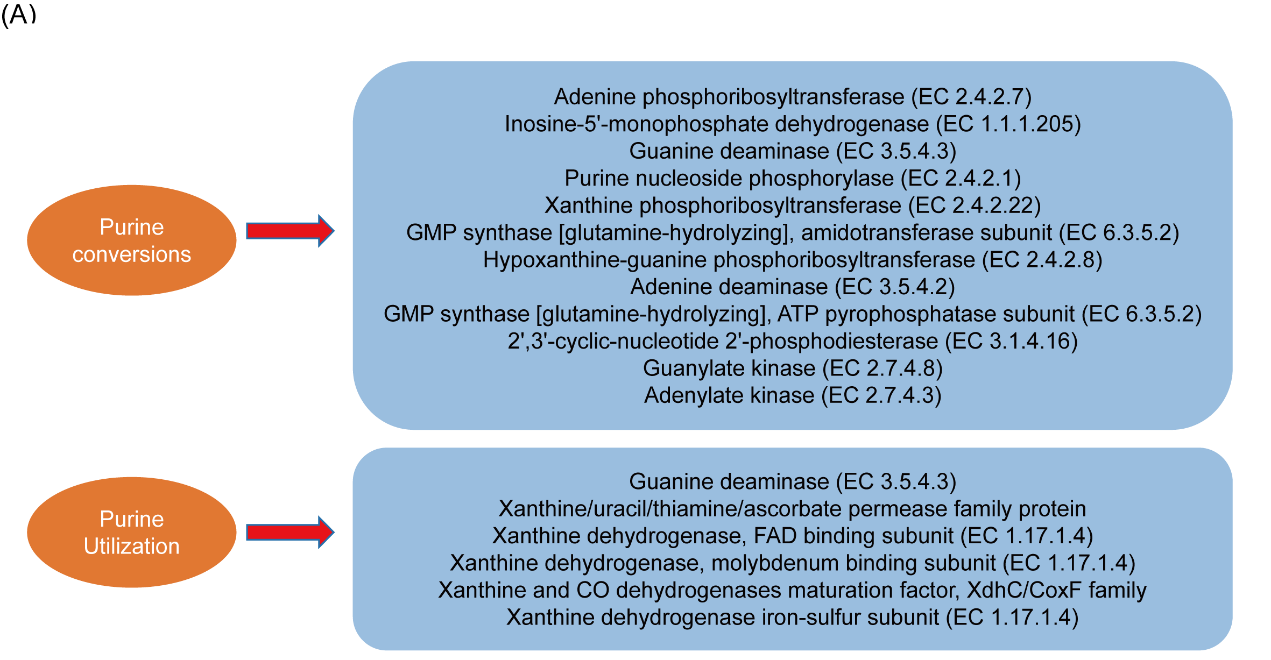


Figure S5. Purine-metabolism related functions of *Phil1 sp001940855*. (A) Enzymes associated with purine metabolism in *Phil1 sp001940855*.
